# Reabilitação Cardíaca Baseada em Tecnologia e Capacidade de Exercício na Doença Arterial Coronariana: Uma Análise de Inteligência Artificial

**DOI:** 10.36660/abc.20260179

**Published:** 2026-06-16

**Authors:** Benil Nesli Ata

**Affiliations:** 1 Ministry of Health Izmir City Hospital Department of Physical Medicine and Rehabilitation Izmir Turquia Ministry of Health, Izmir City Hospital, Department of Physical Medicine and Rehabilitation, Izmir – Turquia

**Keywords:** Reabilitação Cardíaca, Doença da Artéria Coronariana, Inteligência Artificial, Exercício Físico, Saúde Digital

Li com grande interesse o artigo de Saklica et al. intitulado "O impacto da reabilitação cardíaca baseada em tecnologia na capacidade de exercício e na adesão de pacientes com doença arterial coronariana: uma análise com suporte de inteligência artificial", publicado recentemente nos Arquivos Brasileiros de Cardiologia. O estudo aborda um tema importante e oportuno ao examinar o papel potencial de programas de reabilitação cardíaca com suporte tecnológico e explorar a integração de análises baseadas em inteligência artificial (IA) na avaliação dos resultados da reabilitação.^1^ Considerando a persistente subutilização global de programas de reabilitação cardíaca, estudos que exploram estratégias de reabilitação inovadoras e acessíveis são de clara importância clínica.

Um aspecto metodológico que merece maior atenção é a avaliação da capacidade de exercício. No presente estudo, a melhora funcional foi avaliada utilizando o teste de caminhada incremental (TCI). Embora o TCI seja um teste de campo prático e validado, frequentemente utilizado em contextos de reabilitação, ele permanece uma medida submáxima e dependente do esforço. Em pacientes com doença arterial coronariana, o teste cardiopulmonar de exercício (TCPE) é geralmente considerado o padrão de referência para avaliar a capacidade de exercício, pois fornece parâmetros fisiológicos objetivos, como o pico de consumo de oxigênio e os limiares ventilatórios. A inclusão de variáveis derivadas do TCPE poderia, portanto, ter oferecido uma avaliação mais abrangente das adaptações cardiopulmonares e fortalecido a interpretação das melhorias observadas na capacidade funcional.^2^

Outra questão metodológica diz respeito à avaliação da adesão ao exercício entre os grupos de estudo. Nos grupos de intervenção, a adesão foi monitorada por meio de sessões supervisionadas por terapeutas ou registros em aplicativos, enquanto no grupo controle, baseou-se em diários de exercícios preenchidos pelos próprios participantes. Sabe-se que as medidas de atividade física autorrelatadas são suscetíveis a viés de memória e superestimação sistemática, o que pode influenciar a precisão das estimativas de adesão.^3^ Portanto, as diferenças observadas na adesão entre os grupos podem refletir, em parte, variações nos métodos de mensuração, e não diferenças comportamentais reais. O uso de monitoramento de adesão padronizado e objetivo em todos os grupos fortaleceria a validade das comparações em futuros ensaios de reabilitação cardíaca.

Outro aspecto que merece maior discussão é a interpretabilidade clínica das análises baseadas em IA empregadas no estudo. O uso de processamento de linguagem natural e análise de sentimentos para avaliar o feedback dos pacientes representa uma adição metodológica inovadora à pesquisa em reabilitação cardíaca. No entanto, a tradução desses resultados derivados de IA em informações clinicamente acionáveis permanece menos clara. Na pesquisa em saúde digital, se enfatiza cada vez mais que as ferramentas de IA não devem apenas identificar padrões em grandes conjuntos de dados, mas também fornecer resultados que possam informar de forma significativa a tomada de decisões clínicas e o manejo do paciente. Esclarecer como os insights gerados por IA se relacionam com comportamentos de adesão, resultados funcionais ou estratégias de reabilitação individualizadas fortaleceria ainda mais a relevância clínica dessa abordagem tecnológica promissora.^4^

Apesar dessas considerações, o estudo de Saklica et al.^1^ fornece informações valiosas sobre o papel crescente das tecnologias digitais na reabilitação cardíaca e destaca o potencial de programas com suporte tecnológico para aumentar o engajamento e a acessibilidade dos pacientes. Abordar aspectos metodológicos, como avaliação fisiológica abrangente, monitoramento padronizado da adesão e integração clínica mais clara de análises baseadas em IA, pode fortalecer ainda mais futuras investigações nessa área. A continuidade das pesquisas que integram tecnologias de saúde digital com estratégias de reabilitação baseadas em evidências pode contribuir para ampliar o alcance e a eficácia dos programas de reabilitação cardíaca em todo o mundo.

## References

[1] Saklica D, Vardar-Yagli N, Saglam M, Yuce D, Ates AH, Yorgun H (2025). The Impact of Technology-Based Cardiac Rehabilitation on Exercise Capacity and Adherence in Patients with Coronary Artery Disease: An Artificial Intelligence Analysis. Arq Bras Cardiol.

[2] Guazzi M, Arena R, Halle M, Piepoli MF, Myers J, Lavie CJ (2018). 2016 Focused Update: Clinical Recommendations for Cardiopulmonary Exercise Testing Data Assessment in Specific Patient Populations. Eur Heart J.

[3] Prince SA, Cardilli L, Reed JL, Saunders TJ, Kite C, Douillette K (2020). A Comparison of Self-Reported and Device Measured Sedentary Behaviour in Adults: A Systematic Review and Meta-Analysis. Int J Behav Nutr Phys Act.

[4] Topol EJ (2019). High-Performance Medicine: The Convergence of Human and Artificial Intelligence. Nat Med.

