# Supplementary material for: Reabilitação Cardíaca Baseada em Tecnologia e Capacidade de Exercício na Doença Arterial Coronariana: Uma Análise de Inteligência Artificial
Source: Arq Bras Cardiol. 2026 Jun 16;123(5):e20260179. [Article in Portuguese] doi: 10.36660/abc.20260179 (PMC13399085; doi:10.36660/abc.20260179)
Supplement: Translation

# Translation: Technology-Based Cardiac Rehabilitation and Exercise Capacity in Coronary Artery Disease: An Artificial Intelligence Analysis

**DOI:** 10.36660/abc.20260179i

**Authors:** Benil Nesli Ata

**Affiliations:** 1 Izmir Türkiye Ministry of Health, Izmir City Hospital, Department of Physical Medicine and Rehabilitation, Izmir – Türkiye

**Keywords:** Cardiac Rehabilitation, Coronary Artery Disease, Artificial Intelligence, Exercise, Digital Health

I read with great interest the article by Saklica et al. entitled "The Impact of Technology-Based Cardiac Rehabilitation on Exercise Capacity and Adherence in Patients with Coronary Artery Disease: An Artificial Intelligence Analysis," recently published in *Arquivos Brasileiros de Cardiologia*. The study addresses an important and timely topic by examining the potential role of technology-supported cardiac rehabilitation programs and exploring the integration of artificial intelligence (AI)–based analyses in the evaluation of rehabilitation outcomes.^1^ Considering the persistent global underutilization of cardiac rehabilitation programs, studies exploring innovative and accessible rehabilitation strategies are of clear clinical importance.

One methodological aspect that warrants further consideration is the assessment of exercise capacity. In the present study, functional improvement was evaluated using the incremental shuttle walk test (ISWT). Although the ISWT is a practical and validated field test frequently used in rehabilitation settings, it remains a submaximal and effort-dependent measure. In patients with coronary artery disease, cardiopulmonary exercise testing (CPET) is generally regarded as the reference standard for evaluating exercise capacity because it provides objective physiological parameters such as peak oxygen uptake and ventilatory thresholds. The inclusion of CPET-derived variables could therefore have offered a more comprehensive evaluation of cardiopulmonary adaptations and strengthened the interpretation of the observed improvements in functional capacity.^2^

Another methodological issue concerns the assessment of exercise adherence across study groups. In the intervention groups, adherence was monitored through therapist-supervised sessions or application-based records, whereas in the control group, it relied on self-reported exercise diaries. Self-reported physical activity measures are known to be susceptible to recall bias and systematic overestimation, which may influence the accuracy of adherence estimates.^3^ Therefore, the observed differences in adherence between groups may partly reflect variations in measurement methods rather than true behavioral differences. The use of standardized and objective adherence monitoring across all groups would strengthen the validity of comparisons in future cardiac rehabilitation trials.

Another aspect that merits further discussion is the clinical interpretability of the AI–based analyses employed in the study. The use of natural language processing and sentiment analysis to evaluate patient feedback represents an innovative methodological addition to cardiac rehabilitation research. However, the translation of these AI-derived outputs into clinically actionable information remains less clearly defined. In digital health research, it is increasingly emphasized that AI tools should not only identify patterns in large datasets but also provide outputs that can meaningfully inform clinical decision-making and patient management. Clarifying how AI-generated insights relate to adherence behaviors, functional outcomes, or individualized rehabilitation strategies would further strengthen the clinical relevance of this promising technological approach.^4^

Despite these considerations, the study by Saklica et al.^1^ provides valuable insights into the expanding role of digital technologies in cardiac rehabilitation and highlights the potential of technology-supported programs to enhance patient engagement and accessibility. Addressing methodological aspects such as comprehensive physiological assessment, standardized adherence monitoring, and clearer clinical integration of AI–based analyses may further strengthen future investigations in this field. Continued research integrating digital health technologies with evidence-based rehabilitation strategies may contribute to expanding the reach and effectiveness of cardiac rehabilitation programs worldwide.
